# Supplementary figures and images for: Bochum Assessment of Avoidance-based Emotion Regulation for Children (BAER-C): Development and evaluation of a new instrument measuring anticipatory avoidance-based emotion regulation in anxiety eliciting situations
Source: PLoS One. 2023 Jan 13;18(1):e0279658. doi: 10.1371/journal.pone.0279658 (PMC9838827; doi:10.1371/journal.pone.0279658)

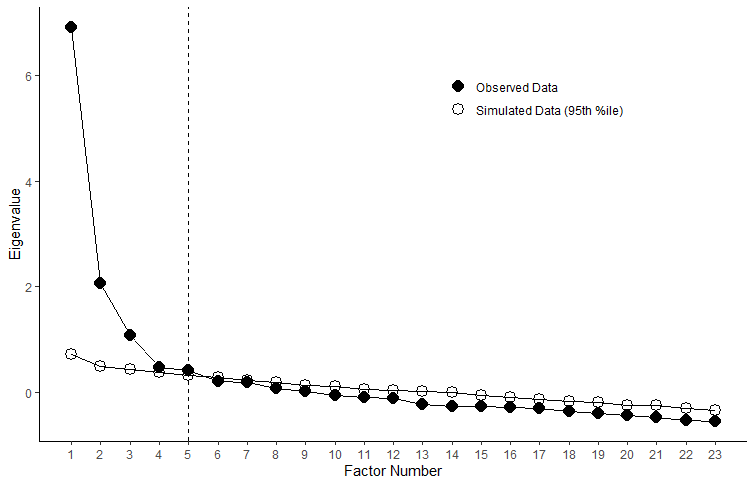


**S1 Figure 1: Scree plot of the Parallel-Analysis suggesting a five-factor solution.**

Supplement: S1 Fig — (DOCX) [file pone.0279658.s001.docx]
