# Supplementary material for: Bochum Assessment of Avoidance-based Emotion Regulation for Children (BAER-C): Development and evaluation of a new instrument measuring anticipatory avoidance-based emotion regulation in anxiety eliciting situations
Source: PLoS One. 2023 Jan 13;18(1):e0279658. doi: 10.1371/journal.pone.0279658 (PMC9838827; doi:10.1371/journal.pone.0279658)
Supplement: S1 Table — (DOCX) [file pone.0279658.s002.docx]

**Supporting Information**

**S1 Table 1.** **Inter-Factor correlations of the BEAR-C subscales.**

|  | 1  Reappraisal | 2  Behavioural Avoidance | 3  Social reassurance | 4  Suppression | 5  Verbal  Reinsurance |
| --- | --- | --- | --- | --- | --- |
| 1  Reappraisal | 1 |  |  |  |  |
| 2  Behavioural Avoidance | .31 | 1 |  |  |  |
| 3  Social reassurance | .33 | .47 | 1 |  |  |
| 4  Suppression | .67 | .46 | .37 | 1 |  |
| 5  Verbal Reinsurance | .56 | .37 | .49 | .53 | 1 |
