# Supplementary material for: Bochum Assessment of Avoidance-based Emotion Regulation for Children (BAER-C): Development and evaluation of a new instrument measuring anticipatory avoidance-based emotion regulation in anxiety eliciting situations
Source: PLoS One. 2023 Jan 13;18(1):e0279658. doi: 10.1371/journal.pone.0279658 (PMC9838827; doi:10.1371/journal.pone.0279658)
Supplement: S2 Table — (DOCX) [file pone.0279658.s003.docx]

**S1 Table 2**: **Additional results of the factor analysis as well as simulated Eigenvalues of the parallel analysis**

| Factor | Eigenvalue of original data | Eigenvalue of simulated data (parallel analysis) | Cumulative Explained Variance |
| --- | --- | --- | --- |
| 1 | 6.92 | 0.59 | .16 |
| 2 | 2.07 | 0.44 | .28 |
| 3 | 1.07 | 0.38 | .37 |
| 4 | 0.47 | 0.32 | .47 |
| 5 | 0.42 | 0.28 | .54 |
| 6 | 0.20 | 0.23 | .57 |
