# Supplementary material for: Bochum Assessment of Avoidance-based Emotion Regulation for Children (BAER-C): Development and evaluation of a new instrument measuring anticipatory avoidance-based emotion regulation in anxiety eliciting situations
Source: PLoS One. 2023 Jan 13;18(1):e0279658. doi: 10.1371/journal.pone.0279658 (PMC9838827; doi:10.1371/journal.pone.0279658)
Supplement: Attachment — Submitted filename: MWLippertetal_BAERC_PLOSOne_Revision_RESPONSETOREVIEWER2.docx [file pone.0279658.s004.docx]

Dear Prof. Olino, dear Reviewer,

Thank you very much for this fair and thorough review. In my opinion your review helped a lot to improve the manuscript and therefore our questionnaire. I hope we have answered all your comments satisfyingly. Please be aware that, following the reviewer’s comments, we redid all analyses splitting the two samples, so the manuscript with tracked changes might be rather confusing. We look forward to hearing your opinion and your feedback on the revised manuscript and are thankful for your help in improving our work.

Editor comments

I also have some queries about the measure. I did not see a description of the number of response options or the degree of skew in the indicators. If the items are rather skewed, that could explain some of the poor model fit.

Answer: Thank you for noticing this. There is a description of the response scale in the methods sections (lines 162,163). We have added the results from the skewness (lines 344ff) analysis to the results section and added a part to the limitations regarding model fit (lines 468ff).

Relatedly, there are estimation options available that provide model fit information from EFAs (including within lavaan). Thus, the transition from CFA to EFA does not fully remove the need to report complete model fit information.

Answer: Thank you for this helpful comment. We have added model fit information for the EFA to the results section. (lines 338,339)

5. Review Comments to the Author

Please use the space provided to explain your answers to the questions above. You may also include additional comments for the author, including concerns about dual publication, research ethics, or publication ethics. (Please upload your review as an attachment if it exceeds 20,000 characters)

Reviewer #1: The present study represents an important potential advance in theoretically driven measurement instruments for emotion regulation, and the instrument shows promise. However, the introduction would benefit from more clarity about the narrow focus of the measure, and more detail about the EFA and CFA need to be provided in the results.

The most significant concerns are around the combination of two separate samples and the lack of validity evidence for the measure. Specific feedback follows.

Introduction

* It would be helpful to include examples of behavioral avoidance, safety behavior, and thought suppression when they are mentioned in the introduction

Answer: Thank you for this comment. I have included some examples in the introduction. (lines 90ff)

* The title of the questionnaire is confusing because it implies avoidance is separate from emotion regulation but the questionnaire was developed along the theory that avoidance is part of the process of emotion regulation. I appreciate the goal of the measure though, to develop a more appropriate assessment of the Gross process model, as my opinion is that the current Gross questionnaire (focusing on reappraisal and suppression) does not adequately assess this model! This has always bothered me, so I really appreciate these authors' approach. That said it is a specific aspect being tested here, avoidance as it pertains to the process model, so however the title of the questionnaire could potentially capture that would be best. I'm not sure why avoidance strategies as they pertain to response modulation were excluded, but again, as long as it's made clear throughout that this measure pertains to anticipatory strategies only, it's not necessarily an issue. Also, to narrow it down even further, the questionnaire just assesses regulation of anxiety specifically! It's going to be tough to capture this very specific narrow approach in the title of the measure so I'm not sure how to address all this. "Avoidance-based anticipatory emotion regulation strategies for anxiety scale for children" is more than a little clumsy!

Maybe a short title for the measure but with an explanatory clause in the title of the article would help (e.g., '______': A measure of ______ for children).

Answer: Thank you for this thoughtful comment. After reconsidering all your argumentation, we agree that the original title might have been confusing. We have changed the title in accordance with your comment to “Bochum Assessment of Avoidance-based Emotion Regulation for Children (BAER-C):Development and Evaluation of a new instrument measuring anticipatory avoidance based emotion regulation in anxiety eliciting situations”. In this way we can keep our acronym while still emphasizing the actual content of the questionnaire and follow your suggestion.

Method + results:

Answer: Thank you for your thorough review and all your comments on the methods and results. We apologize for the confusion of having two samples integrated into one. We understand that having two samples in which not all participants filled out the same questionnaires is problematic and highly confusing. To solve this, we have decided to restructure our study and redo all calculations. To solve the sample problem, we have separated the samples. In a first step, we have used the CAD sample to do an exploratory factor analysis, like you suggested. We also did item analyses as well as an analysis of internal consistency and parts of construct validity using all the questionnaires we have in the CAD sample. In a second step, we used the student sample to confirm the factor structure found in the CAD sample with a CFA. We also used the FEEL-KJ data for additional information on construct validity. In a third step, we compared both samples for criterion validity. Because of the new factor analysis, which only used the CAD sample, two additional items were removed because of low factor loadings. That leads to different results in the MANCOVA comparing the samples (e.g., no interaction effect group*gender). Therefore, the methods and especially the results have changed considerable.

* It's unclear why these extra items were generated "In addition, items concerning the subjective skill to handle anxiety and the acceptance of anxiety were generated and all but one were worded without negations" as well as the extra 30th item. Were these items included in the analyses?

If so, which items were they?

Answer: The extra items were generated following an exploratory approach as an addition to the model, focusing on two other ER approaches (acceptance and the subjective feeling to be able to control emotions). Due to the item analysis these items were removed even before the factor analysis because of low discriminatory power or because the lowered overall internal consistency. (lines 313ff)

* It's strange that the CFA was just mentioned in passing in the method section rather than given a full section in the results with all the appropriate information provided.

Answer: Thank your noticing this. In the new structure, the use of EFA and CFA is clearer (see overall comment for methods and results).

* The differences in administration and characteristics between the two samples are a little concerning. Ideally the samples would be analyzed separately and assessed for any differences between them, or at least this would be done and noted in a footnote if no differences were found.

Answer: Thank you for pointing this out. We agree that the use of two samples combined into one was rather confusing. Following your suggestion, we have redone the analyses with separate samples (see overall comment for methods and results).

* Were all participants given potential reading assistance or only in the second sample where it is noted?

Answer: Yes, in both groups, assistance was offered. I have changed the wording, so that it is clearer for the reader. (line 206)

* The confirmatory factor analysis is mentioned again in passing in the statistical analysis section, and described as unsuccessful--this is confusing, and once again, more detail should be provided about these analyses (at the very least, more of the fit statistics, and an indication of exactly what model was tested). A little more detail is provided in the results section but it's unclear each time it's mentioned previously that it will be discussed later in the paper and even when it comes up in the results section more information should be provided. It's an unusual strategy to do the CFA first and then do an EFA when the CFA fails. Usually an EFA would be conducted first and the CFA to confirm the structure that was uncovered. Although the measure was developed based on theory, it still seems the traditional analytic process would make more sense. The CFA should also generally be done in a separate sample than the EFA. So, ultimately, it might make more sense to leave the CFA out of this paper entirely and conduct a follow up study to confirm the EFA structure in a new sample.

Answer: Thank you, once again, for pointing out the problem with the factor analysis. Please see the overall comment for the methods and results.

Results

* More information should be provided on the EA in the determining of the number of factors - numeric results from the parallel analysis, the scree plot, the Eigenvalues and % of variance explained, etc.

Answer: Thank you for this recommendation. I have added numeric details of the parallel analysis, as well as Eigenvalues of the data and explained variance per factor to the manuscript. As far as I know this is usually not reported in detail in the manuscript, so I have added the information to the electronic supplement. If you would like to see the table in the actual manuscript, I would be glad to move it. (ESM table 2)

* Exactly how much would the overall reliability decline if item 1 was removed, because ideally items with low factor loadings should not be retained?

Answer: In the new analysis item 1 was removed because of a low factor loading.

* It's unclear whether item 9 was retained on both subscales.

Answer: In the new analysis item 9 only loaded satisfyingly on one subscale.

* Item 23 does not seem to make sense on the reappraisal subscale and may need to be removed

Answer: It seems the children interpreted the item differently than we have thought. After discussing this in the author team we have decided to keep it on the reappraisal subscale. The children in the CAD group seem to have interpreted the item as that they ignore what makes them afraid (and think of something positive instead). We have emphasized the discrepancy in the discussion and see need for further research here. (lines 452ff)

* Although it states in text this information is in Table 2, I don't see the factor intercorrelations in the table.

Answer: Thank you for noticing this mistake. The information on factor intercorrelations can be found in the electronic supplements

* The lack of correlations with the SDQ internalizing should be mentioned under the convergent validity header (as that was their intention), not divergent. Thus, convergent validity is only partially confirmed. Similarly, the correlations with adaptive subscale should be listed under divergent validity. There is a problem with the divergent validity evidence, as there is not evidence of divergent validity for this scale.

Answer: Thank you for bringing this to our attention. In the new analysis the internalizing and externalizing as well as adaptive and maladaptive scale correlations are labeled correctly as convergent or divergent validity.

* It's unclear why some analyses were done with the samples separated and some were not.

Answer: Thank you for this comment. In the new analysis this should be clearer (see overall comment for methods and results).

* It seems like it should say p < .001, not p > .001.

Thank you for noticing this mistake. I have corrected the wrong symbols.

Discussion

* Unfortunately, I would not agree that the construct has been confirmed, given the poor divergent evidence. Ultimately though, the biggest issue is there are not enough validity measures in the study, so it's difficult to draw conclusions. There should be validity measures that are more other existing emotion regulation and strategy questionnaires, not just validity measures of psychopathology. It's one thing to show relations to externalizing problems where none were expected - as the authors state, ER is a transdiagnostic factor that is related to a lot of pathology - however, avoidance should be less related to externalizing, so it's still concerning. However, the correlations with adaptive coping are more concerning, given the lack of other relations to other ER measures among which to contextualize this.

Answer: Thank you for bringing this to our attention. We have added these concerns in the discussion. The new results are similar regarding the adaptive ER strategies correlations. We have reframed the results and limited it, especially for construct validity. Nonetheless, the results for factor structure and reliability, as well as some parts of construct and criterion validity are promising. We have tried to bring this discrepancy to the reader’s attention and emphasized a need for more research to clarify this, especially with other ER questionnaires (e.g., lines 540ff)

* The authors should interpret why the security scale possibly ended up splitting into two separate factors.

Answer: Thank you for this helpful comment. We think that the factor split up in a more active and a more passive component of situation modification. We have added a sentence to the discussion. (lines 461ff)

* There should be a more thorough discussion of limitations.

Answer: Thank you for noticing this. In accordance with the new results, we have completely reworked the limitations. (lines 525ff)

Thank you once again for this fair and thorough review. It helped us a lot to improve our work.
