# Supplementary material for: Bochum Assessment of Avoidance-based Emotion Regulation for Children (BAER-C): Development and evaluation of a new instrument measuring anticipatory avoidance-based emotion regulation in anxiety eliciting situations
Source: PLoS One. 2023 Jan 13;18(1):e0279658. doi: 10.1371/journal.pone.0279658 (PMC9838827; doi:10.1371/journal.pone.0279658)
Supplement: Attachment — Submitted filename: MWLippertetal_BAERC_PLOSOne_Revision2_RESPONSETOREVIEWER.docx [file pone.0279658.s005.docx]

Dear Prof. Olino, dear Reviewer,

Thank you once again for taking the time to review our manuscript. We deeply appreciate your recognition of the effort put into the revision so far. We have, once again, tried to answer and solve each of your comments. We hope all changes are to your satisfaction.

Reviewer Comments

Although the items were later dropped, it’s still not clear why these additional items (subjective skill, acceptance of anxiety) were generated since they don’t fit with the model. This information should be provided because the reader doesn’t know yet when they are mentioned that they will later be dropped, so it’s still confusing to read. Another idea could be to relegate this information to a footnote later on so it doesn’t take on such a high priority when describing the scale – but even in that case more information should be provided about why these items were developed.

Thank you for this remark. We have added information and literature to the method section that explains these items a bit more. (lines 180ff)

- The statement about “for scoring…” that is on page 10 line 197 of the tracked changes copy should be moved to after the results section because we can’t determine how the questionnaire should be scored until the factor structure information is presented.

Thank you for this helpful comment, of course, this is correct! We have moved the paragraph to the end of the factor analysis section. (lines 379ff)

- For validity, I would refer to it as construct validity, not criterion validity (as it’s not being compared to a gold standard measure of the same construct). Also, it would be useful to provide validity information in both samples separately (and then together, if desired).

Thank you for this comment. Criterion validity was changed to construct validity throughout the manuscript. The results regarding the validity are presented separately for each sample, only the SCAS was used in both samples. To avoid confusion regarding the use of the questionnaires in one sample or both samples, we have chosen not to present data of both samples combined.

- Low reliability of SDQ scales should be mentioned in the limitations as a potential issue with attenuating correlations.

Thank you for this comment to further improve the manuscript. A sentence was added to the discussion section. (lines 542f)

- Please provide examples of the maladaptive and adaptive strategies on the FEEL-KJ. Considering that in the results the measure was correlated positively with both adaptive and maladaptive scales, it would be helpful to contextualize why that might be by knowing more about what distinguishes the items.

Thank you for noting this. We have added the subscales to the method section. (lines 317ff)

- On line 378, I’m not sure why it says the differences between the samples were used to ‘check for criterion validity’.

Thank you for this helpful clarification. It was changed to construct validity.

- What is meant by three items were removed because of low discriminatory power? The way the results section reads, this happened before the factor analysis, so is this based on something other than factor loadings?

This is correct. Before exploratory factor analysis items were analyzed for item difficulty and discriminatory power. Those three items were removed because discriminatory power was <.30.

- More specific information about how much each removed item lowered the overall consistency (or at least what it was before and after the three items were removed) should be provided.

Thank you for pointing this out. The alpha value increased from .88 to .91. In the analysis we acted conservatively and dropped any item, which would increase alpha. Due to the dropping of the overall alpha value following your other comment, we have included those items in the factor analysis. All three items dropped out because they failed to load on any of the five factors. We have changed this in the results section. (lines 373f)

- It is my experience that eigenvalue and % of variance information is generally presented in the manuscript itself, so that’s ideally where I would like to see it – but I’ll defer to the editor if they feel this material is best suited for supplementary materials.

- Thank you for once again clarifying this. Dear Editor, would it be possible to clarify where you think this data should be in the manuscript?

- In the eigenvalue table, the values for factor 6 should be provided so that it’s clear the simulated data eigenvalue is higher than the original at that point.

- Thank you for this remark. Data for the 6^th^ factor was added to the manuscript.(Table ESM 2)

- The scree plot should be provided.

- The scree plot was added to the electronic supplements.

- The eigenvalue data is not necessarily all pointing to the same conclusion – which is not to say that I disagree with the five factor solution, especially in light of the parallel analysis and the % of variance explained – but it’s also noteworthy that there are three factors with eigenvalues above 1, and there’s a huge drop between factors 1 and 2 (though hard to tell in proportion without the scree plot), so a 1 or 3 factor solution would also be defensible. These potential solutions and the decision making process justifying going with the five factor solution should be described in text.

Thank you for this helpful comment. We have added additional explanations to the discussion section of the paper, discussing possible one and three factor solutions. (lines 506ff)

- The loadings for item 1 and 25 should be provided even though they were removed (and can be italicized or otherwise indicated in the table if necessary for clarity).

- The loadings were added to the table and following your suggestion were italicized. (Table 2)

- There shouldn’t be model fit statistics TLI and RMSEA provided for an exploratory factor analysis.

Thank you for this comment. The editor asked for TLI and RMSEA values for the exploratory analysis. So, I would keep it in the hands of the editor if those values should be removed.

- It isn’t indicated that the authors intend users to create an overall total score across all 21 items, so if this doesn’t make conceptual sense, then an overall alpha shouldn’t be provided across all 21 items (or if it is intended, this should be clarified).

Thank you for this remark to clarify the manuscript. We have deleted the overall Alpha value but have kept the Omega as this uses the factor structure of the EFA. We think the results are much clearer now.

- Is the validity with the SCAS calculated combining both samples? Since the other measures are only in one sample or the other, this measure should be analyzed separately for each sample.

- Thank you for pointing this out. The SCAS validity was calculated using the anxiety sample. We have not reported the correlation for the student sample so far. We have added it to the table with the FEEL-KJ correlations (see table 5)

- Do the SCAS-C and SDQ go in opposite directions, in terms of how they’re scored? Please clarify in the methods section how they’re coded because in Table 4 it’s unclear why the social reassurance scale is correlated in opposite directions with the SCAS-C and the SDQ internalizing…and also whether the correlations with suppression and reappraisal indicate higher levels of externalizing problems.

- Both questionnaires are scored similarly (from 0 to 2 and from 0 to 3) so we think this does not explain the correlation. Nonetheless we have added the information to the method section (lines 278 & 297.).

- In the divergent validity section, it should also say that social reassurance did not correlate significantly with externalizing symptoms.

- Thank you for this comment. We have changed the paragraph in the divergent validity section (line 419).

- Please also report the chi square value for the CFA model.

- The chi square value was added to the paper. The chi square test is significant. Following the recommendations of and Alavi and colleagues (2020) referring to Cole (1987) we have calculated the ratio ChiSqaure/df as it is more reliable regardless of the sample size. The ratio is 1.54 and thus below 2, therefore showing acceptable model fit especially in combination with the other values. We have added all information to the paper, as we have neglected them so far to keep the paper shorter (lines 427ff).

- The loadings in the CFA should also be reported, either in text, a table, or a figure, whatever is preferable. A figure would be ideal so the structure can be visualized. Were the intercorrelations between the factors modeled (since they were accounted for in the EFA model)?

Thank you for the remark. A figure visualizing the model was added to the results section. The intercorrelations were accounted for in the CFA model as well.

- The comparison between the two samples (3.7) should be presented earlier, before the rest of the results – probably when the rest of the sample characteristics are presented. Section 3.8 should stay where it is.

- Thank you for this clarifying comment. We have moved the section to the methods section right after the sample characteristics. (lines 236ff)

- I think there’s a typo in line 567 “trying that someone is with the child” – maybe there’s a word missing?

- Thank you for notifying. We have corrected the sentence. (line 525)

- In the discussion when the CAMS is referenced, it’s unclear how that’s relevant to a specific discussion of internal consistency.

- Thank you for this comment. We have clarified the paragraph that was supposed to show that the internal consistency of the BAER-C is comparable to other questionnaires in the field. (lines 529f)

- The statement in the discussion that all avoidance strategies except behavioral were associated with externalizing symptoms does not appear consistent with the results.

Thank you for the remark. The part in the discussion was corrected to match the result section and once again suggesting problems especially with the suppression subscale. (lines 556ff)
