# Supplementary material for: Bochum Assessment of Avoidance-based Emotion Regulation for Children (BAER-C): Development and evaluation of a new instrument measuring anticipatory avoidance-based emotion regulation in anxiety eliciting situations
Source: PLoS One. 2023 Jan 13;18(1):e0279658. doi: 10.1371/journal.pone.0279658 (PMC9838827; doi:10.1371/journal.pone.0279658)
Supplement: Attachment — Submitted filename: reivew PLUSONE 2.docx [file pone.0279658.s006.docx]

**Dear Authors,**

Thank you for designing this precious study.

1. That is suggested to provide a short description for “anxiety disorders” in first paragraph in INTRODUCTION section.
2. It should be better change “ criterion validity” to “ construct validity”.
3. Please mention examples of the maladaptive and adaptive strategies on the FEEL-KJ.
4. Please more explain why removed three items.
5. chi square value for the CFA model is not reported!

Sincerely
