# Supplementary material for: Bochum Assessment of Avoidance-based Emotion Regulation for Children (BAER-C): Development and evaluation of a new instrument measuring anticipatory avoidance-based emotion regulation in anxiety eliciting situations
Source: PLoS One. 2023 Jan 13;18(1):e0279658. doi: 10.1371/journal.pone.0279658 (PMC9838827; doi:10.1371/journal.pone.0279658)
Supplement: Attachment — Submitted filename: MWLippertetal_BAERC_PLOSOne_Revision3_RESPONSETOREVIEWER.docx [file pone.0279658.s007.docx]

Dear Dr. Nazari, dear Reviewers,

Thank you once again for taking the time to review our manuscript. We deeply appreciate your recognition of the effort put into the revision so far. We have, once again, tried to answer and solve each of your comments and to respect all author guidelines. We hope all changes are to your satisfaction.

Editor Comments

First: Please, follow the Author guideline.
■ in text citation, use Square bracket [ ], with numbers.
■ add Doi to references.

All references have been updated and changed. I apologize for overlooking.

Unnecessary use of prepositions at the beginning or between sentences has reduced the writing quality of the article. I suggest you that consult with professional editor.

Thank you for your thorough work and this helpful comment. As recommended a copy editor from the field of clinical psychology re-checked the manuscript. We have removed or rewritten all sentences in which the prepositions could be removed without changing the meaning of the sentence. We think the manuscript is a lot tighter now.

Following issues are Minor. However, some items are critical.
1. Add legend for tables and figure.

All legends for tables and figures have been adapted to author guidelines and example manuscript. Table 3 was intentionally left without legend.

2.The abstract is not informative. Add some statisticall results.

Thank you for this helpful comment, I have added some statistical results to the abstract.

3. Please follow IMRAD. the limitations is correct. Outlook should be deleted.

Thank you for noting that detail. Outlook is deleted.

4. Please, speak about generalizability.

Thoughts about generalizability were added to the discussion. (lines 570-577)

5. The study should be written according to " standard checklist". ● this item is critical. Please attach the STROBE CHECKLUST.

The STROBE Checklist was attached and filled out according to the STROBE article. Thank you for bringing this to our attention. The Checklist really helped to tighten the structure of the paper. Very few points were not applicable because of the structure of this study introducing a new questionnaire.

6. The figure should be presented in standard form.
The current figure is not scientific.

We have changed the figure according to the journal guidelines.

Reviewer 1 Comments

The authors have been very responsive to the reviews, and I think the scale will make an important contribution to the literature. I believe the paper should be accepted for publication, with the following last few minor suggestions for improvement:

The information justifying the acceptance and subjective skill is good, but the end of the paragraph is still confusing. It still says four subscales with 29 items were created (and then a 30th item), so it’s not clear where the acceptance and subjective skill items went. It looks like the 4 subscales covered 25 items, based on the tables – so I guess I would put the statement ‘four subscales with 25 items were created’ before the information about acceptance and subjective skills, and then state that an additional four items were added to cover these topics.

Thank you for this helpful comment to further clarify the manuscript. I have followed your suggestions and changed the paragraph according to it. (lines 173 ff)

On line 185 of the tracked changes copy, the word importance doesn’t seem correct. I’m not sure what word is supposed to be there, but that word doesn’t make sense.

Thank you for pointing this out, The mistake was corrected.

You note that validity is examined separately, but on line 216 it states “Finally, to check construct validity both patient and community sample were combined.” I think you mean to say validity was examined in both the patient and community samples?

Thank you once again. I have removed the misleading sentence, as the process was explained better in the paragraph already.

If the validity correlations presented really are for different samples depending on the measure, it should be made more clear which sample each result is coming from (stated in the results when each set of results is presented).

Thank you for this remark. I have clarified this in the headings of the paragraphs. (lines 394, 411)

When I said it wasn’t clear how testing differences between the samples would speak to validity, I meant it should be made more clear what the expectation is and how it would speak to validity (e.g., if scores were higher in the clinical sample this would indicate that the scale accurately measured avoidance based emotion regulation). And actually, that one could be considered criterion validity! I think you can change that particular instance back (or leave it, whatever you prefer).

Thank you for clarifying. I think I leave it the way it is now as it is clear in its structure.

The explanation added to the discussion regarding choosing the five factor solution over the one or three factor solution should be in the second paragraph of the results section instead.

Following your recommendations, the paragraph was moved to the results section. Thank you.

The CFA figure is very helpful, thank you! One suggestion would be to make the oval for the reappraisal figure bigger, and spell out that word, so that it’s clear the reappraisal and avoidance factors are on the same hierarchical level and correlated with each other (I do see the double headed arrow but the size and orientation make it easy to miss) rather than reappraisal appearing as another lower level factor under avoidance.

Thank you for this comment. The figure was changed according to the editors comments. I have tried to include your feedback as well.

Reviewer 2 Comments

Thank you for designing this precious study.
1. That is suggested to provide a short description for “anxiety disorders” in first paragraph in INTRODUCTION section.

Thank you for your suggestion. I have added a very short DSM-5 TR definition to the introduction section. (lines 54f)

2. It should be better change “criterion validity” to “ construct validity”.

I have checked the whole manuscript and was not able to find that “criterion validity” was still in use. I would kindly ask you, to tell me the lines, in which you have found “criterion validity”.

3. Please mention examples of the maladaptive and adaptive strategies on the FEEL-KJ.

Paragraph 2.5.4 describes the questionnaire and lists all strategies which load on the adaptive and the maladaptive second order factor. Please tell me, if you would like example for the strategies in other paragraphs as well. To keep the paper short, I would prefer to leave it in this paragraph.

4. Please more explain why removed three items.

Thank you for this remark. The items were removed because their discriminatory power was too low (below the threshold of .30). This is stated in the first section of the results.

5. chi square value for the CFA model is not reported!

Thank you for this remark. The χ²-value was added to the results section.
